# Personal Cold Protection Behaviour and Its Associated Factors in 2016/17 Cold Days in Hong Kong: A Two-Year Cohort Telephone Survey Study

**DOI:** 10.3390/ijerph17051672

**Published:** 2020-03-04

**Authors:** Holly Ching Yu Lam, Zhe Huang, Sida Liu, Chunlan Guo, William Bernard Goggins, Emily Ying Yang Chan

**Affiliations:** 1 National Heart and Lung Institute, Imperial College London, Emmanuel Kaye Building, London SW3 6LR, UK; ching.lam@imperial.ac.uk; 2 Collaborating Centre for Oxford University and CUHK for Disaster and Medical Humanitarian Response (CCOUC), Faculty of Medicine, The Chinese University of Hong Kong, Shatin, Hong Kong SAR, China; huangzhe@cuhk.edu.hk (Z.H.); kevin.liu@cuhk.edu.hk (S.L.); theresachunlanguo@gmail.com (C.G.); 3 Jockey Club School of Public Health and Primary Care, Chinese University of Hong Kong, New Territories, Hong Kong, China; wgoggins@cuhk.edu.hk; 4 Nuffield Department of Medicine, University of Oxford, Oxford OX3 7LF, UK

**Keywords:** cold, personal health protective behaviour, associated factors, risk perception, subtropical city

## Abstract

*Background:* Despite larger health burdens attributed to cold than heat, few studies have examined personal cold protection behaviours (PCPB). This study examined PCPB during cold waves and identified the associated factors in a subtropical city for those without central heating system. *Methods*: A cohort telephone survey was conducted in Hong Kong during a colder cold wave (2016) and a warmer cold wave (2017) among adults (≥15). Socio-demographic information, risk perception, self-reported adverse health effects and patterns of PCPB during cold waves were collected. Associated factors of PCPB in 2017 were identified using multiple logistic regression. *Results:* The cohort included 429 subjects. PCPB uptake rates were higher during the colder cold wave (*p* < 0.0005) except for ensuring indoor ventilation. Of the vulnerable groups, 63.7% had low self-perceived health risks. High risk perception, experience of adverse health effects during the 2016 cold wave, females and older groups were positive associated factors of PCPB in 2017 (*p* < 0.05). *Conclusions:* PCPB changed with self-risk perception. However vulnerable groups commonly underestimated their own risk. Indoor ventilation may be a concern during cold days in settings that are less prepared for cold weather. Targeted awareness-raising promotion for vulnerable groups and practical strategies for ensuring indoor ventilation are needed.

## 1. Introduction

Low ambient temperatures are associated with adverse health effects such as hypothermia and higher risk of cardiovascular diseases, respiratory diseases and infectious diseases globally [1,2,3,4,5]. The elderly, people with illnesses and outdoor workers are more vulnerable during conditions of extreme temperatures [3]. In warmer regions, although winters may be considered milder than in colder regions, due to the less appropriate housing design for low temperatures and acclimatization [4], effects of unusual low temperature might increase mortality and morbidity. Previous studies have shown that populations residing in lower latitudes were more vulnerable to cold temperature [5] and had higher threshold temperatures at which cold effects began to be observed [1]. Adverse health effects related to low temperatures and cold waves have been reported in subtropical regions including Hong Kong, Guangzhou, China, Taiwan and Brisbane, Australia [4,6,7,8,9,10,11,12,13]. 

As highlighted in the Sendai Framework [14], understanding risks and enhancing preparedness are priorities to support bottom up risk reduction and resilience in communities. Despite the scientific evidence showing the adverse health effects of extreme low temperatures [1,15,16], studies examining personal cold protection behaviours (PCPB) are rare [17]. Several studies focusing on heat protective measures and associated factors have been published, probably due to raising concerns about increasing global temperature [18,19,20,21,22]. Studies from temperate regions have shown socio-demographic factors such as sex and economic status were associated with the uptake of personal protection measures against extreme temperatures [17,18]. In the United Kingdom (UK), females and people with higher income were more likely to apply personal heat protection measures during heat waves [18]. A European study also found females were more likely to wear more outdoor clothes on cold days but the insulation of clothes was poorer than those of males [17]. 

Risk perception is another important determinant of health behaviours [23,24] Studies from the UK [19], China [20] and Pakistan [21] have reported positive associations between risk perception and protective behaviour against high temperatures. Vulnerable groups, however, had been reported more likely to underestimate their health risks during extreme high temperatures [25]. A focus group study from New York City, United States, found that seniors and people with fair or poor health conditions were not aware of their own risk during hot days [22]. 

Despite the expected increase in number of hot days and average temperature [26], cold effects on human health are, however, still more severe than the effects of heat and should not be neglected [1,4]. Individual cold protection behaviour, risk perception and other potential associated factors, such as experience of adverse health effects in previous cold waves and socio-demographic factors, are mostly unknown and make the formulation of evidence-based cold-related health protection policy and promotion challenging. 

This two-year telephone survey cohort study, conducted immediately after an extreme cold wave in 2016 and a regular cold wave in 2017: (1) explored the perceived health risks and risk perception accuracy at low temperatures; (2) examined the pattern of PCPB; and (3) identified the associated characteristics of PCPB in a subtropical city. The results of this study will support the facilitation of drafting health protection strategy to reduce avoidable health risk during cold waves in warmer regions. 

## 2. Materials and Methods

### 2.1. Study Period

In Hong Kong, there is no clear definition of a cold wave. The Hong Kong Observatory takes into consideration multiple meteorological parameters and issues a cold weather warning signal when cold weather may incur harm to the public. In this study, cold waves are defined as periods when cold weather warning signals were issued by the Hong Kong Observatory.

This is a two-year telephone survey-based cohort study. Two telephone surveys were conducted, during February of 2016 and March of 2017, one week after the cold weather warning signals were issued by the Hong Kong Observatory on 21–27 January 2016 and 23–27 February 2017, respectively. The surveys were conducted shortly after the cold waves to reduce recall bias. The 2016 survey was completed in eight days while the 2017 survey was finished in eleven days. The cold wave in 2016 was severe and 24 January 2016 was the coldest day in Hong Kong since 1957. The average daily mean temperature during the 2016 cold wave was 10.6 °C (average daily mean temperature in January in 1981, 2010: 16.3 °C). The 2017 cold wave was relatively milder in intensity. The average daily mean temperature within the study cold wave period was 14.8 °C (average daily mean temperature in February in 1981, 2010: 16.8 °C). 

#### Sampling and Subject Recruitment

A random digit dialling approach was used to select households from a full list of landline telephone numbers in Hong Kong and the last birthday method, inviting the household member with birthday closest to the interview date, was used to select Cantonese speaking subjects ≥ 15 years old for the baseline survey in 2016. Quota sampling was adopted to match the population characteristics in terms of age-group, gender and residential districts in the Hong Kong SAR Census in 2011 (2016 Census information was not available at the time of the baseline study). The baseline sample size of 1000 was based on being able to estimate the percentage of people applying a particular cold protection measure with maximum margin of error of 3.5% at a 95% confidence level. Phone calls were made in the evening on weekdays and throughout the day on weekends to minimize bias based on employment status. Oral consent had been sought from each participant at the beginning of the surveys. The study was conducted in accordance with the Declaration of Helsinki and the protocol was approved by the Survey and Behavioural Research Ethics Board, The Chinese University of Hong Kong. 

### 2.2. Variables

Socio-demographic characteristics, self-report history of chronic diseases (conditions that require medical treatment for more than six months), health risk perceptions of cold weather, self-reported health outcomes and protection behaviour patterns within the study period were collected in the survey. Based on the health guidelines provided by the Hong Kong Observatory [27], four personal cold protection measures, which included ‘putting on more clothes’, ‘avoid staying in windy areas’, ‘use of heating equipment’ and ‘ensuring adequate indoor ventilation’, were studied. The possible biological associations between the four behaviours and human health are shown in Table 1. Details of questionnaire design and phone call algorithm for the 2016 data have been published elsewhere [28]. Follow-up surveys were conducted with the same cohort and using same questions after the 2017 cold wave.

#### Measurement of Risk Perception Accuracy

To assess the risk perception accuracy of subjects, the objective risk of subjects was compared with their self-risk perception. Subjects fulfilling at least one of these four factors in 2017, old age (≥60 years), history of chronic diseases, living alone and receiving comprehensive social security assistance (CSSA), were considered to be at high health risk during low temperatures. This assumes that old age and a history of chronic diseases increase vulnerability physiologically, while living alone and receiving CSSA are related to less resources and support. Underestimation of risk was defined as subjects who fulfilled one or more of these risk factors (the high-risk group) but did not consider themselves high risk. Considering that there might be other risk factors of cold related health problems not included in this study, people who appeared to be overestimating their risk (reported themselves as high risk but fulfilled none of the four pre-set criteria) were grouped as “potentially overestimated”. Subjects from the high-risk group who considered themselves high risk, and those who did not have any of the pre-set criteria and considered themselves low risk were defined as having correct perception. 

### 2.3. Statistical Method

Chi-square test was used to compare the uptake rate of the PCPB between the two cold waves. Associated factors for the uptake of protective behaviours in 2017 cold wave were identified using multiple logistic regression models. A two-stage model selection approach, univariate analyses (chi-square test) followed by multiple logistic regression, was adopted. Factors with *p* < 0.2 in univariate analyses were entered a logistic regression for examining the independent associations in the second stage. Chi-square test was performed to examine the characteristics of the lost-to-follow-up group. All analyses were performed using IBM SPSS 21(IBM, Armonk, NY, USA).

### 2.4. Model Selections in Identifying Associated Factors of PCPB

Socio-demographic factors such as age, sex and income have been reported to be associated factors of heat protective behaviours [17,18]. Age-group, gender, household income and residential district were therefore included in all regression models as the core model. Other independent variables considered in the model selection process included education level, CSSA status, occupation, marital status, history of chronic diseases, risk perception, risk perception accuracy, feeling cold at home during the 2017 cold period and experience of adverse health effect during the 2016 cold waves that needed medical treatment or medicine. Dependent variables, the uptake of the four PCPBs during the 2017 cold wave (Yes/ No), were examined separately.

## 3. Results

### 3.1. Descriptive Results

A total of 1017 subjects were recruited for the 2016 baseline survey and 429 of these subjects have completed the follow-up survey in 2017 (follow-up rate 42.2 %). A diagram demonstrating the subject recruitment process is shown in Supplementary Figure S1.

Descriptive statistics of the personal characteristics of the 2016–2017 cohort are shown in Table 2 with the comparison to the characteristics of the Census. Although the baseline sample was comparable to the 2011 census characteristics, it should be noted that the proportion of the elderly ≥65 years old in the follow-up sample in 2017 was higher than that in the baseline, due to the lower follow-up rate among the working population (25–44 years) (lost-to-follow-up analysis available in Supplementary Table S2).

#### 3.1.1. Risk Perception and Risk Perception Accuracy

In the 2017 survey, 45.0% of the subjects fulfilled at least one of the pre-defined risk factors and were considered as high risk in low temperatures. Overall, 23.3% of subjects considered themselves having higher health risk during low temperature (Table 2). Regarding risk perception accuracy, 62.2% had correct risk perception, 28.7% had underestimated their risk and 7.5% had potentially overestimated the risk. Among the high-risk group, 63.7% (123/193) had underestimated their risk. 

#### 3.1.2. PCPB Patterns

**Figure 1:**
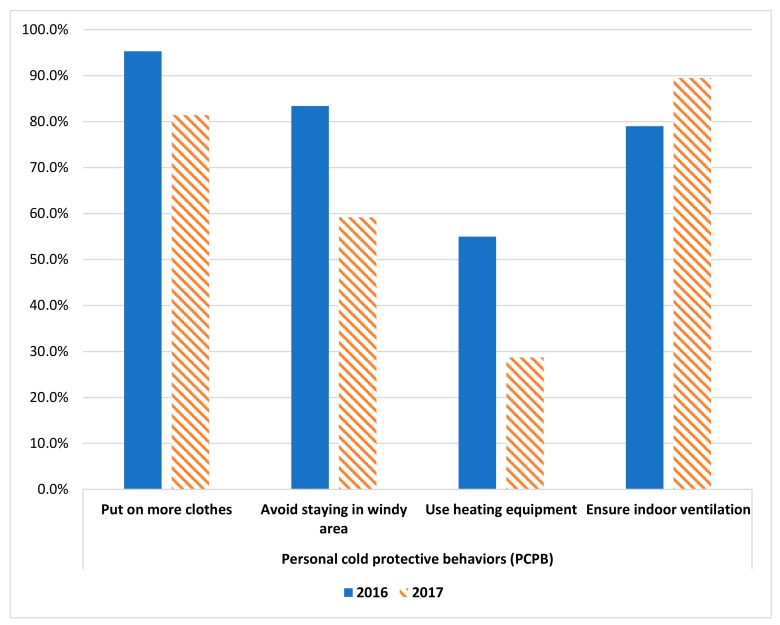
Uptake rate of personal cold protection behaviour among the same group of subjects in 2016 and 2017 cold wave (*n* = 429). Remarks: *p*-values of Chi-square test comparing the four personal cold protective behaviours between 2016 (the colder cold wave) and 2017 (a warmer cold wave) are all <0.0005.

In general, warm-keeping PCPB uptake rate was statistically significantly higher during the colder cold wave in 2016 (*p* < 0.0005 in chi-square test) (Figure 1). During the colder cold wave in 2016, 95.3% had reported putting on more clothes, 83.4% had avoid staying in windy area and 55.0% has used heating equipment during the cold wave (80.2 % among those owning heating equipment). In the milder cold wave in 2017, 81.4% had reported putting on more clothes, 59.2% had avoided staying in windy area and 28.7% had used heating equipment (41.0 % among those owning heating equipment). In contrast, the proportion of subjects that had ensured indoor ventilation increased from 79.0% in 2016 to 89.5% in 2017. 

### 3.2. Associated Factors of PCPB

The selected distribution of PCPB across levels of covariates and the respective chi-square test results are presented in Table 3. A full table of results of all variables considered can be found in Supplementary Table S1. Subjects with previous experience of adverse health effect in the 2016 cold wave were more likely to consider themselves as high risk at low temperature (*p*-value for chi-square test = 0.003). To avoid multicollinearity, self-risk perception was excluded from multiple logistic regression models whenever previous experience of adverse health effect was included in the model, and vice versa.

#### 3.2.1. Put on More Clothes

Those who lived in Kowloon (compared to those who lived on Hong Kong Island) (Odds Ratios (OR) (95% confidence interval) 2.64 (1.15 to 6.04)), those who had experienced adverse health effects during the 2016 cold wave (5.48 (1.26 to 23.83)) and those who perceived high health risk at low temperatures (2.30 (1.03 to 5.16)) were more likely to put on more clothes (Table 4). 

#### 3.2.2. Avoid Staying in Windy Area

Compared to residents from the Hong Kong Island, those living in Kowloon (2.18 (1.20 to 3.97)) and the New Territories (2.65 (1.50 to 4.67)) were more likely to avoid staying in a windy area. Age group 40-59 (2.15 (1.03 to 4.50)) were also more likely to stay away from the wind compared to the youngest age-group 15–24. 

#### 3.2.3. Use Heating Equipment

Females (1.85 (1.14 to 3.00)), those who felt very cold at home (3.72 (1.29 to 10.72)), the older age-groups (vs. 15–24) and those who had high health risk perception (2.57 (1.48 to 4.44)) were more likely to use heating equipment during the cold wave. The ORs of using heating equipment for the older age-groups (vs. 15–24) ranged from 3.31 to 4.00 (Table 4). In subgroup analysis among those who owned any heating equipment at home by age, feeling cold at home remained statistically significant.

#### 3.2.4. Ensure Indoor Ventilation

The elderly aged above 60 years (ORs range from 7.22 to 14.57), those who lived in Kowloon (3.07 (1.16 to 8.16)) and were married (3.33 (1.34 to 8.28)) were more likely to ensure indoor ventilation. 

## 4. Discussion

In summary, 45.0% of the subjects were considered under high health risk during cold weather but more than 60% (28.7% of all subjects) of this vulnerable group had underestimated their health risk. The uptake rates of cold protective measures were generally higher in a stronger cold wave except for ensuring indoor ventilation. Regarding associated factors of PCPB, those who had experienced adverse health effects during the 2016 cold wave, who perceived high health risk in low temperatures, females and the older groups (≥60) were more likely to apply PCPB in the cold waves studie. 

Risk perception was associated with warm-keeping, cold protective behaviours in this study (wearing more clothes and using heating equipment). This was consistent with the results from previous hot effect studies from the UK [19], China [20] and Pakistan [21]. However, a significant proportion of subjects did not consider themselves more vulnerable in extremely low temperatures regardless of their age and history of chronic diseases and similar results have been reported in UK [38] and North American based cities (proportions not reported) [39]. The findings did highlight the gap in health literacy and self-risk perception and reconfirmed the need for targeted health education and services for the vulnerable groups, such as people with chronic disease and the elderly, to reduce exposures, and also highlighted the corresponding health outcomes and the related health burden. The two-year survey-based study has also suggested the adaptation ability of the population to low temperature by implementing personal protective measures based on personal experiences.

Our study found that females were more likely to uptake cold protective behaviours which agreed with the findings from previous studies [17,18]. Previous studies suggested this may be associated with less willingness to seek care or help among men than women, as reported previously [40]. Targeted health promotion can be considered for the male group. Older age was another demographic factor associated with higher adoption of PCPB, which was contradictory to the previous hot effect studies in the UK [18] and New York City [22]. Although the effect of age on uptake of health protection behaviours is unclear, the differences in physiological conditions that affect homeostatic process between age groups may explain our result. Older people generally have lower metabolic rate and compromised thermoregulation [41,42], which may make the elderly more sensitive to cold than heat [43]. Income or CSSA status, which was associated with hot protective behaviour in the UK [18], and education level, which was associated with cold protective behaviour in Europe, were not found to be associated in this study. One possibility is that most of the PCPBs in this study are straight forward, well-promoted by the Hong Kong Observatory and financially affordable (e.g., avoid staying in windy area), which made them less likely to affected by income and education level.

Special attention should be paid to results for ensuring indoor ventilation. While the population were more likely to adopt warm-keeping measures in the colder winter (2016), they were less likely to manage indoor ventilation. Hong Kong is a subtropical city in which central heating and housing design for cold insulation are not common. On cold days, people tend to shut doors and windows to reduce the drop in indoor temperature. Unlike regions with colder climates, facilities enhancing ventilation, such as trickle vent on window frames, are rarely found in Hong Kong. Shutting doors and windows leads to poor indoor ventilation. Poor ventilation is associated with higher risk of infectious diseases [4]. Indoor air quality and heating have been raised as an important element in building a sustainable living environment [44]. More investigation is needed to seek practical solutions in balancing indoor warming and ventilation during cold days in different settings to reduce relevant health risks.

To the authors’ knowledge, this is the first study examining personal cold protective behaviours in a setting without central heating systems and cold-insulation housing design. This study covered PCPB on the coldest day in the region in the past six decades which allowed us to capture PCPB in response to an unusual cold wave and compare it to a that in a normal cold wave. The sample was representative in terms of gender and residential districts. The immediate outreach to subjects after the cold waves also helped reduce recall bias. This study has several limitations. Although the land-based telephone list covered 94% of fix line telephones in Hong Kong, the households that were not in the list of land-based telephone service were not included [28]. The follow-up rate was low due to the length of the survey (about 30 minutes). Thus, the age distribution of the 2017 follow-up sample might not be comparable to that of the general population. We adjusted age-group in statistical models for identifying associated factors of PCPB to reduce the bias. Due to the relatively small sample size, all chronic diseases were grouped and assessed as a single variable which might also introduce bias as behaviour may vary by disease. Effects of microclimate that might affect personal protective behaviours were not included in this study. The prompt interviews conducted, starting one week after the cold waves, might not be able to capture all adverse health effects, as cold-related health effects tended to have long lags [45,46,47]. The proportion of reporting unwell might be underestimated.

## 5. Conclusions

Our results showed that PCPB changed according to the intensity of cold waves, age, gender, past experiences and risk perception. Study findings agree with previous studies that vulnerable groups commonly underestimated their health-risk which might deter PCPB and increase risk of adverse health effects. Targeted health promotion should be provided to vulnerable groups, such as those with chronic diseases, old aged and living alone, to increase risk perception, and to males to raise their awareness of health protection against cold to reduce avoidable cold-related health risks. This study also raised the concern in balancing indoor warming and ventilation in warmer regions that are less prepared for low temperature. Studies investigating warm-keeping solutions without compromising ventilation should inform cold-related health protection strategies.

## Figures and Tables

**Table 1:** Biological association between personal cold protective behaviours and health outcomes.

Protective Measure	Linkage with Human Health	Related Health Benefits from Literature	Personal Characteristics Associated with the Behaviour from Literature
Wearing More clothes	Prevent heat loss through insulation and resistance to evaporation, wind and water. Inner layer to control body temperature and humidity, middle layer for insulation and outer layer to protect against the outer environment [29].	Control heat loss, insulate current temperature and reduce discomfort due to cold injury and hypothermia. Increase manual working performance. High moisture absorbing material can keep the skin dry even when sweating. Ventilating garments prevent post-chilling effect when the wet garment is drying [29].	Elderly people in the UK with problems such as thyroid, poor circulation, anaemia and heart irregularities wore more clothes. To supplement appliance to keep warm [30], Japanese female cooperative workers were more likely to wear one or more items of clothing [31].
Avoid Windy Areas	Protect wind chills from reducing skin temperature through rapid evaporation, especially when weather is overcast. [32]	Reduce the risk of hip fractures, incidence of asthma, sickle cell disease and acute pain. [33]	Hong Kong Observatory released advice to “avoid prolonged exposure to wintry winds.” [34] No literature was found to evaluate the local population’s wind-related behaviour.
Use of Heaters	Maintaining adequate indoor temperature. [35]	Increase resistance to respiratory and vascular complications, such as myocardial infractions. Improvement in symptoms of asthma in children and reduced time off school. [8,36]. Ensure thermoregulatory function in elderly people, as the minimum indoor temperature for them should be a few degrees higher than average. [35]	Availability of heating system(s) such as heaters, fireplace, central heating, etc. [30]
Ensure Indoor Ventilation	Intended to remove pollutants emitted from indoor sources, e.g., building materials, furnishings, unvented combustions, etc.	Associated with reduced prevalence of sick building syndrome and allergic manifestation in children. Literature suggests low ventilation rates are associated with inflammation and respiratory infections [37].	A few elders in the UK opened windows for air circulation, mostly for a short while. Most considered it potentially wasteful of heat. [30] Some elders opened windows at night to sleep and turned off the heater [35].

Remark: Protective measures included were references from the Hong Kong Observatory.

**Table 2:** Descriptive statistics of socio-demographic variables, history of chronic diseases and health perception of subjects in 2017 follow-up survey, with comparison to the major socio-demographic characteristics in Hong Kong Census 2016 and 2016 baseline sample.

Demographics	HK 2016 Population By-Census Data (*n* = 6,506,130)		Sampled Participants in 2016 (*n* = 1017)		Follow-Up Participants in 2017 (*n* = 429)		2017 Sample vs. Census *p*-Value ^a^
	*n*	%	*n*	%	*n*	%	
Gender							1 ^b^
Male	2,947,073	45.3%	437	43.0%	194	45.20%	
Female	3,559,057	54.7%	580	57.0%	235	54.80%	
Age							<0.001
15–24	785,981	12.1%	126	12.4%	49	11.40%	
25–44	2,228,566	34.3%	315	31.0%	109	25.40%	
45–64	2,328,430	35.8%	384	37.8%	165	38.50%	
≥65	1,163,153	17.9%	192	18.9%	106	24.70%	
Geographical distribution *							0.43
Hong Kong Island	1,120,143	17.2%	182	17.9%	83	19.30%	
Kowloon	1,987,380	30.6%	315	31.0%	133	31.00%	
New Territories	3,397,499	52.2%	518	51.0%	213	49.70%	
Education attainment							<0.01
Primary and below	1,673,431	25.7%	137	13.5%	59	13.80%	
Secondary	2,841,510	43.7%	501	49.4%	215	50.10%	
Post-secondary	1,991,189	30.6%	377	37.1%	154	35.90%	
Marital status							0.34 ^b^
Single	2,708,709	41.6%	410	40.5%	188	43.80%	
Married	3,797,421	58.4%	602	59.5%	239	55.70%	
Monthly household income (HKD)							
<10,000					67	15.60%	
10,000–19,999					72	16.80%	
20,000–29,999					72	16.80%	
30,000–39,999					60	14.00%	
40,000 or above					128	29.80%	
Comprehensive social security assistant (CSSA)							
Yes					17	4.00%	
No					412	96.00%	
Occupation							
Clerical					66	15.40%	
Non clerical					108	25.20%	
Housewife					88	20.50%	
Student					37	8.60%	
Retired/ unemployed					123	28.70%	
History of chronic diseases							
Yes					113	26.30%	
No					316	73.70%	
Living alone							
Yes					38	8.90%	
No					388	91.40%	
Own heating equipment at home							
Yes					252	58.70%	
No					175	40.80%	
Felt cold at home during the study cold period in 2017							
Very cold					24	5.60%	
Cold					253	59.00%	
Not cold					149	34.70%	
Reporting any adverse health effects during the 2016 cold waves (needed medical treatment or medicine)							
Yes					49	11.40%	
No					380	88.60%	
Self- risk perception in low temperature							
High					100	23.30%	
Low					324	75.50%	
Risk at low temperature based on pre-defined factors							
High					193	45.00%	
Low					234	54.50%	
Health perception accuracy ^c^							
Correct					267	62.2%	
Underestimated					123	28.7%	
Potentially overestimated					32	7.5%	

^a^ χ2 test was used to measure the overall difference in proportions between this survey and the 2016 Hong Kong Population Census data. *p*-value < 0.05 indicates significant difference. ^b^ χ2 test with continuity correction was used. ^c^ Underestimated: People fulfilled at least one of the four preset criteria (old age, history of chronic diseases, living alone and receiving CSSA) but did not consider themselves high risk. Correct: People fulfilled at least one of the four preset criteria and considered themselves high risk AND People did not have any of the preset criteria and considered themselves low risk. Overestimated: People did not have any of the preset criteria but considered themselves high risk. * Marine population are excluded. Remarks: Percentage may not add up to 100% due to missing data.

**Table 3:** Association between personal characteristics and uptake of personal cold protection behaviour in 2017 cold wave using Chi-square test.

Personal Characteristics	Put on More Cloths			Avoid Staying at Windy Area			Use Heating Equipment			Keep Indoor Ventilation		
	No	Yes	* *p*-Value	No	Yes	* *p*-Value	No	Yes	* *p*-Value	No	Yes	* *p*-Value
Gender												
Male	39	155	0.37	89	104	0.03	155	39	<0.0005	21	173	0.84
	50.0%	44.4%		51.4%	40.9%		50.7%	31.7%		46.7%	45.1%	
Female	39	194		84	150		151	84		24	211	
	50.0%	55.6%		48.6%	59.1%		49.3%	68.3%		53.3%	54.9%	
Age												
15–24	10	39	0.64	21	28	0.09	42	7	0.08	13	36	<0.0005
	12.8%	11.2%		12.1%	11.0%		13.7%	5.7%		28.9%	9.4%	
24–39	18	65		36	47		58	25		11	72	
	23.1%	18.6%		20.8%	18.5%		19.0%	20.3%		24.4%	18.8%	
40–59	22	117		45	94		98	41		18	121	
	28.2%	33.5%		26.0%	37.0%		32.0%	33.3%		40.0%	31.5%	
60–69	18	68		35	52		64	23		2	85	
	23.1%	19.5%		20.2%	20.5%		20.9%	18.7%		4.4%	22.1%	
≥70	10	60		36	33		44	27		1	70	
	12.8%	17.2%		20.8%	13.0%		14.4%	22.0%		2.2%	18.2%	
Education												
Primary or below	8	51	0.46	28	30	0.29	41	18	0.78	3	56	0.06
	10.3%	14.6%		16.2%	11.9%		13.4%	14.8%		6.7%	14.6%	
Secondary	38	176		80	134		157	58		19	196	
	48.7%	50.4%		46.2%	53.0%		51.3%	47.5%		42.2%	51.2%	
Post-secondary or above	32	122		65	89		108	46		23	131	
	41.0%	35.0%		37.6%	35.2%		35.3%	37.7%		51.1%	34.2%	
Residential districts												
Hong Kong Island	17	65	0.05	48	35	0.001	64	19	0.15	12	71	0.41
	21.8%	18.6%		27.7%	13.8%		20.9%	15.4%		26.7%	18.5%	
Kowloon	15	117		53	80		87	46		12	121	
	19.2%	33.5%		30.6%	31.5%		28.4%	37.4%		26.7%	31.5%	
New Territories	46	167		72	139		155	58		21	192	
	59.0%	47.9%		41.6%	54.7%		50.7%	47.2%		46.7%	50.0%	
Self-risk perception at low temperature												
Low risk	64	259	0.03	137	185	0.18	249	75	<0.0005	34	290	0.89
	86.5%	74.4%		79.7%	74.0%		81.9%	65.5%		75.6%	76.5%	
High risk	10	89		35	65		55	45		11	89	
	13.5%	25.6%		20.3%	26.0%		18.1%	37.5%		24.4%	23.5%	
Risk-perception accuracy												
Correct	50	207	0.12	102	156	0.34	188	70	0.26	28	230	0.05
	68.5%	59.7%		59.6%	62.7%		62.0%	58.8%		63.6%	60.8%	
Under-estimate	22	115		61	75		100	38		10	128	
	30.1%	33.1%		35.7%	30.1%		33.0%	31.9%		22.7%	33.9%	
Potentially over-estimate	1	25		8	18		15	11		6	20	
	1.4%	7.2%		4.7%	7.2%		5.0%	9.2%		13.6%	5.3%	
Experience of adverse health effect in 2016 cold wave and needed medical consultation/ any form of treatment												
No	75	303	0.02	157	221	0.23	278	102	0.02	37	343	0.16
	96.2%	86.8%		90.8%	87.0%		90.8%	82.9%		82.2%	89.3%	
Yes	3	46		16	33		28	21		8	41	
	3.8%	13.2%		9.2%	13.0%		9.2%	17.1%		17.8%	10.7%	

*p-value for Chi-square test.

**Table 4:** Adjusted Odds-ratio (OR) of associated factors of personal cold protective behaviours in 2017 cold wave using multiple logistic regression.

Associated Factor	Personal Cold Protective Measures											
	Put on More Cloths (*n* = 398)			Avoid Staying at Windy Area (*n* = 398)			Use Heating Equipment (*n* = 399)			Keep Indoor Ventilation (*n* = 398)		
	OR	95%CI		OR	95%CI		OR	95%CI		OR	95%CI	
		Lower	Upper		Lower	Upper		Lower	Upper		Lower	Upper
^ Gender												
Male	1.00	-	-	1.00	-	-	1.00	-	-	1.00	-	-
Female	1.08	0.62	1.85	1.34	0.88	2.04	**1.85**	**1.14**	**3.00**	0.92	0.46	1.85
^Age												
15–24	1.00	-	-	1.00	-	-	1.00	-	-	1.00	-	-
25–39	0.86	0.33	2.23	1.22	0.57	2.62	**3.31**	**1.12**	**9.77**	1.74	0.64	4.73
40–59	1.70	0.66	4.35	**2.15**	**1.03**	**4.50**	**3.34**	**1.18**	**9.50**	1.12	0.38	3.36
60–69	0.74	0.27	2.06	1.52	0.68	3.41	2.52	0.82	7.74	**7.22**	**1.13**	**46.02**
≥70	1.02	0.32	3.26	0.95	0.39	2.30	**4.00**	**1.210**	**13.19**	**14.57**	**1.33**	**159.55**
^ Residential districts												
Hong Kong Island	1.00	-	-	1.00	-	-	1.00	-	-	1.00	-	-
Kowloon	**2.64**	**1.15**	**6.04**	**2.18**	**1.20**	**3.97**	1.94	0.97	3.91	**3.07**	**1.16**	**8.16**
New Territories	1.15	0.57	2.29	**2.65**	**1.50**	**4.67**	1.44	0.73	2.84	2.10	0.88	5.02
^ Income												
<10000	1.00	-	-	1.00	-	-	1.00	-	-	1.00	-	-
10000–19999	0.70	0.24	2.03	1.50	0.69	3.26	0.51	0.221	1.18	2.02	0.36	11.34
20000–29999	0.66	0.22	1.99	1.14	0.53	2.45	0.73	0.32	1.68	0.77	0.15	3.85
30000–39999	0.42	0.14	1.29	1.20	0.52	2.74	0.63	0.25	1.56	1.22	0.22	6.73
≥40000	0.42	0.15	1.18	1.00	0.47	2.11	0.70	0.31	1.56	1.49	0.29	7.74
^ Marital status												
Currently not married	-	-	-	-	-	-	-	-	-	1.00	-	-
Currently married	-	-	-	-	-	-	-	-	-	**3.33**	**1.34**	**8.28**
^ Felt cold at home during the cold period in 2017												
No	-	-	-	-	-	-	1.00	-	-	-	-	-
Cold	-	-	-	-	-	-	1.24	0.77	2.08	-	-	-
Very cold	-	-	-	-	-	-	**3.72**	**1.29**	**10.72**	-	-	-
^ Experience of adverse health effect in 2016 cold wave and needed medical consultation/ any form of treatment												
No	1.00	-	-	-	-	-	1.00	-	-	-	-	-
Yes	**5.48**	**1.26**	**23.83**	-	-	-	1.89	0.97	3.71	-	-	-
^#^ Health risk perception at low temperatures												
Low	**1.00**	-	-	-	-	-	1.00	-	-	-	-	-
High	**2.30**	**1.03**	**5.16**	-	-	-	**2.57**	**1.48**	**4.44**	-	-	-

ORs were estimated from Logistic Regression models. **Bold**: *p*-value < 0.05 in Logistic Regression. ^ Not adjusted for “Health risk perception at low temperatures”. ^#^ Not adjusted for “Experience of adverse health effect in 2016 cold wave and needed medical consultation/any form of treatment”.

## Supplementary Materials

The following are available online at https://www.mdpi.com/1660-4601/17/5/1672/s1, Table S1: Association between personal characteristics and uptake of personal cold protection behaviour in 2017 cold wave using Chi-square test; Table S2: Table showing age distribution in participants in the lost-to-follow-up group and 2016-17 cohort group; Figure S1: Diagram showing subject recruitment process.
