# Supplementary material for: Personal Cold Protection Behaviour and Its Associated Factors in 2016/17 Cold Days in Hong Kong: A Two-Year Cohort Telephone Survey Study
Source: Int J Environ Res Public Health. 2020 Mar 4;17(5):1672. doi: 10.3390/ijerph17051672 (PMC7084253; doi:10.3390/ijerph17051672)
Supplement: Supplementary file 1 [file ijerph-17-01672-s001.pdf]

International Journal of
Environmental Research and Public Health

MDPI

Supplementary

Table A1. Biological association between personal cold protective behaviours and health outcomes.

Protective Measure	Linkage with human health	Related health benefits from literatures	Personal characteristics associated with the behaviour from literatures
**Wearing More clothes**	Prevent heat loss through insulation and resistance to evaporation, wind, and water. Inner layer to control body temperature and humidity, middle layer for insulation, and outer layer to protect against the outer environment.[1]	Control heat loss, insulate current temperature, and reduce discomfort due to cold injury and hypothermia. Increase manual working performance. High moisture absorbing material can keep the skin dry even when sweating. Ventilating garments prevent post-chilling effect when the wet garment is drying.[1]	Elderly people in the UK with problem such as thyroid, poor circulation, anaemia, and heart irregularities wore more clothes. Also to supplement appliance to keep warm.[2] Japanese female cooperative workers were more likely to wear one or more clothes.[3]
**Avoid Windy Areas**	Protect wind chills from reducing skin temperature through rapid evaporation, especially when there is overcast.[4]	Reduce the risk of hip fractures, incidence of asthma, sickle cell disease, and acute pain.[5]	Hong Kong Observatory released an advisement to "avoid prolonged exposure to wintry winds."[6] No literature was found to evaluate the local population's wind-related behaviour.
**Use of Heaters**	Maintaining adequate indoor temperature.[7]	Increase resistance to respiratory and vascular complications, such as myocardial infractions. Improvement in symptoms of asthma in children and reduce time off school. [8] Ensure thermoregulatory function in elderly people as the minimum indoor temperatures for them should be a few degrees higher than average.[7]	Availability of heating system(s) such as heaters, fireplace, central heating, etc.[2]
**Ensure Indoor Ventilation**	Intend to remove pollutants emitted from indoor sources e.g. building materials, furnishings, unvented combustions etc.	Associated with reduced prevalence of sick building syndrome, allergic manifestation in children. Limited data suggest low ventilation rates are associated with inflammation and respiratory infections.[9]	A few elders in the UK opened windows for air circulation, mostly for a short while. Most considered it potentially wasteful of heat.[2] Some elders opened windows at night to sleep and turned off the heater[7]

Remark: Protective measures included were reference from the Hong Kong Observatory.

Table A2: Table showing age distribution in participants in the lost-to-follow-up group and 2016-17 cohort group.

	Participants that lost-to-follow-up n (%)	Participants in both 2016 and 2017 survey n (%)	Total n (%)	p-value of chi-square test

Age group (year)	15-24	75 (12.8)	49 (11.4)	124 (12.2)	<0.0005
	25-39	141 (24.0)	83 (19.3)	224 (22.0)	
	40-59	250 (42.5)	139 (32.4)	389 (38.2)	
	60-69	71 (12.1)	87 (20.3)	158 (15.5)	
	>=70	51 (8.7)	71 (16.6)	122 (12.0)	
	Total	588	429	1017	

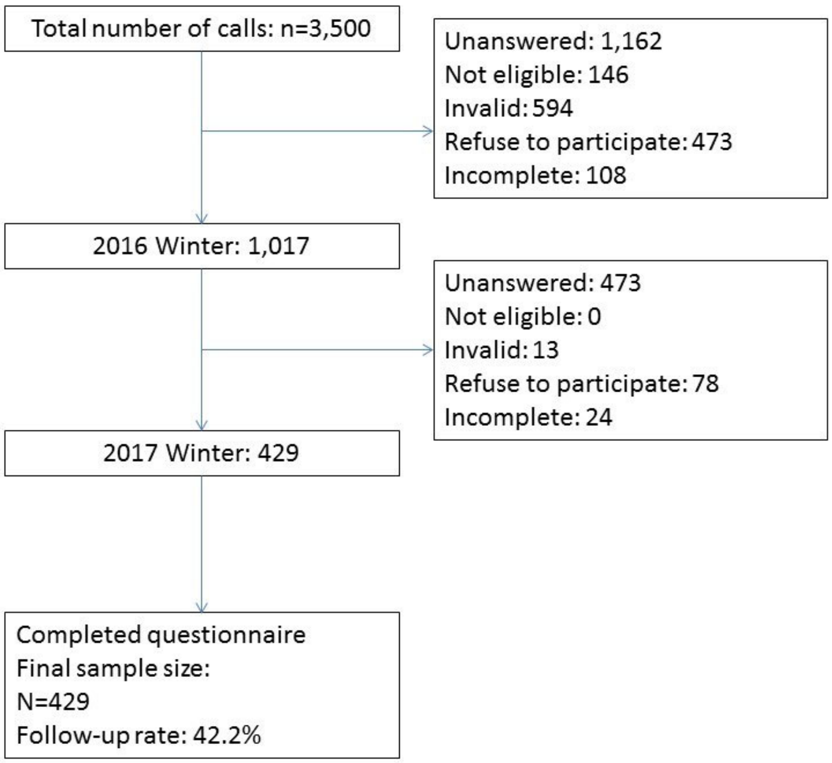

Figure A1. Diagram showing subject recruitment process.

Reference

1 FærevikH.Clothing and protection in arctic environments. 2015;:5–9. doi:10.13140/RG.2.1.1931.1444
2 DayR, HitchingsR.Older people and their winter warmth behaviours: Understanding the contextual dynamics. 2009;:60.
3 TannerLM, MoffattS, MilneEMG, *et al.*Socioeconomic and behavioural risk factors for adverse winter health and social outcomes in economically developed countries: a systematic review of quantitative observational studies. *J Epidemiol Community Health* 2013;**67**:1061–7. doi:10.1136/jech-2013-202693
4 BuddGM.Ergonomic aspects of cold stress and cold adaptation. 2017.
5 JonesS, DuncanER, ThomasN, *et al.*Windy weather and low humidity are associated with an increased number of hospital admissions for acute pain and sickle cell disease in an urban environment with a maritime temperate climate. *Br J Haematol* 2005;**131**:530–3. doi:10.1111/j.1365-2141.2005.05799.x
6 LiP, ChanS.Application of a weather stress index for alerting the public to stressful weather in Hong Kong. *Meteorol Appl* 2000;**7**:369–75. doi:10.1017/S1350482700001602
7 McKeeCM.Deaths in winter: Can Britain learn from Europe? *Eur J Epidemiol* 1989;**5**:178–82. doi:10.1007/BF00156826

8 OlsenND.Prescribing warmer, healthier homes. *Br Med J* 2001;**322**:748–9.
9 SundellJ, LevinH, NazaroffWW, *et al.*Ventilation rates and health: Multidisciplinary review of the scientific literature. *Indoor Air* 2011;**21**:191–204. doi:10.1111/j.1600-0668.2010.00703.x
